# Historical range contractions can predict extinction risk in extant mammals

**DOI:** 10.1371/journal.pone.0221439

**Published:** 2019-09-05

**Authors:** Christielly Mendonça Borges, Levi Carina Terribile, Guilherme de Oliveira, Matheus de Souza Lima-Ribeiro, Ricardo Dobrovolski

**Affiliations:** 1 Instituto de Biologia, Universidade Federal da Bahia, Salvador, Bahia, Brazil; 2 Instituto de Biociências, Universidade Federal de Goiás, Jataí, Goiás, Brazil; 3 Centro de Ciências Agrárias, Ambientais e Biológicas, Universidade Federal do Recôncavo da Bahia, Cruz das Almas, Bahia, Brazil; Institute of Zoology, CHINA

## Abstract

Climate change is amongst the main threats to biodiversity. Considering extant mammals endured Quaternary climate change, we analyzed the extent to which this past change predicts current mammals’ extinction risk at global and biogeographical scales. We accessed range dynamics by modeling the potential distribution of all extant terrestrial mammals in the Last Glacial Maximum (LGM, 21,000 years ago) and in current climate conditions and used extinction risk from IUCN red list. We built General Linear Mixed-Effects Models to test the magnitude with which the variation in geographic range (ΔRange) and a proxy for abundance (ΔSuitability) between the LGM and present-day predicts current mammal’s extinction risk. We found past climate change most strongly reduced the geographical range and climatic suitability of threatened rather than non-threatened mammals. Quaternary range contractions and reduced suitability explain around 40% of species extinction risk, particularly for small-bodied mammals. At global and biogeographical scales, all groups that suffered significant Quaternary range contractions now contain a greater proportion of threatened species when compared to groups whose ranges did not significantly contract. This reinforces the importance of using historical range contractions as a key predictor of extinction risk for species in the present and future climate change scenarios and supports current efforts to fight climate change for biodiversity conservation.

## Introduction

Climate change is expected to cause severe impacts on biodiversity including change in geographical range and local abundance [1–3]. The magnitude of future climate change is a key predictor of species extinction risk and accelerated biodiversity loss [4], and when combined with habitat loss and fragmentation, pollution, introduction of exotic species and overexploitation—or the "evil-quartet" [5], represents one of the main conservation challenges of modern times [6]. Therefore, climate change is increasingly a part of the current biodiversity crisis, in which the observed extinction rates are much higher than expected from historical background rates [7,8], with 22% of global vertebrates, including mammals, listed as threatened by the International Union for Conservation of Nature (IUCN) Red List [9,10].

In response to climate change, species’ geographic ranges displace, expand or contract [11–13]. Extinction risk is frequently related to species' range contractions and such analyses have been widely used to provide comprehensions about species at population and community levels [14]. Current geographic range size is also a strong correlate of extinction risk in mammals, amphibians and birds [15–17], subsequent to IUCN’s Red List Criterion B for Endangered Species [18]. However, we have limited knowledge on the magnitude to which species have undergone historical range contractions or expansions, meaning species currently in small ranges may have had larger, smaller or identical range sizes in the past. Most studies on extinction risk and range contractions focus mainly on the historical post-Columbus period, the last 500 years only [19,20]. Nonetheless, climate dynamics have been modifying natural ecosystems for a longer time through the geological past [21].

The influence of climate change on extinction and extinction risk has been assessed mainly through two approaches: the effect of past climate change on past extinctions, e.g. Quaternary megafauna extinctions [22–24], or the effect of future climate change on extant species [3,4]. Expressive climate changes occurred over the Quaternary glaciations, which combined with human impacts resulted in the extinction of many large mammal species in all continents across the last 50,000 years [25]. Extant mammals withstood Quaternary climate change as well and many species are still dealing with the negative impacts it may have caused them.

Considering past extinction events, we propose that Quaternary climate change, specifically across the last ice age, altered climatic conditions and this change represented shifts in species abundance in time and space. For some species it reduced the favorable climatic conditions with negative effects on their geographic ranges and population demography [22]. Consequently, if species’ ranges contracted during this period it would make them more vulnerable to subsequent human impacts. Thus, we expect historic range dynamics to explain the extinction risk and population decline of extant terrestrial mammals currently listed as threatened with extinction by IUCN.

Here we tested the hypothesis that the Quaternary climate change effect on species range dynamics is correlated to the current extinction risk and population decline in extant mammals. We built models at global and biogeographical scales and for different taxonomic groups. By showing significant relationships between Quaternary range contraction and IUCN’s extinction risk status, our findings support our prediction and indicate that deep past climate changes keep challenging species, making them more vulnerable to current human impacts.

## Material and methods

### Data

Occurrence data for all extant mammals where gathered from the IUCN red list database (http://www.iucnredlist.org). The IUCN red list aims at monitoring the conservation status of species and is the most comprehensive inventory of the global conservation status of biological species. We searched for native terrestrial mammals according to their biogeographic realm and obtained their extent of occurrence. We converted the extent of occurrence (given as polygons) into occurrence points at a resolution of 0.5° of latitude/longitude. Point data was later used to model mammals’ range in both the Pre-industrial (present-day) and the Last Glacial Maximum (LGM; 21,000 year ago) periods. From a total of 4645 species, 793 are listed in one of the IUCN’s threat categories: Critically Endangered (CR), Endangered (EN) and Vulnerable (VU), 3390 are listed as Near Threatened (NT) and Least Concerned (LC), and 462 species are listed as Data Deficient (DD) (Table A in S1 File).

Climatic data represented by 19 bioclimatic variables were obtained for the present-day and LGM periods from the ecoClimate database (http://ecoclimate.org) at a resolution of 0.5° latitude/longitude. All climate layers were obtained for four Ocean Atmospheric General Circulation Models (OAGCM): CCSM4, CNMR, GISS-E2-R and MIROC-ESM. To avoid collinearity amongst climate predictors when building ENMs, we applied a varimax-rotated factor analysis and selected the variable with the highest loading in each one of the first five rotated factors [26]. Climate predictors were selected for each realm and resulted in five bioclimatic variables for the Afrotropic, Neotropic and Palearctic and four for Australasia and Indomalaya (Table B in S1 File).

### Ecological niche modeling

To access Quaternary range dynamics, we modeled the ecological niche and potential distribution of all extant terrestrial mammals for the present-day and LGM period following the ensemble approach [27]. For this, we used 13 methods: BIOCLIM, Ecological Niche Factor Analysis (ENFA), Euclidian Distance (ED), Gower Distance (GD), Mahalanobis Distance (MD), Genetic Algorithm for Rule Set Production (GARP), Generalized Linear Models (GLM), Maximum Entropy (Maxent), Generalized Additive Models (GAM), Flexible Discriminant Analysis (FDA), Multivariate Adaptive Regression Splines (MARS), Neural Networks (ANN) and Random Forest (RNDFOR). Because some methods require absence data, we selected, randomly, pseudo-absences and background across all realms' grids keeping prevalence at 50%. For methods that do not need pseudo-absence, we used the pseudo-absence as background [28].

Species’ ENMs were built in each separated biogeographic realm to make results comparable among species and to constraint the selection of pseudo-absences/background into the species realm. We randomly selected 75% of the presence and pseudo-absence data to calibrate the models and the remaining 25% to evaluate them. This procedure was repeated 50 times [26] and models performance was evaluated with True Skill Statistics (TSS; [29]). Models with poor performance (TSS < 0.5) were initially eliminated and ensemble computed by weighted averaging the suitabilities from remaining good models using TSS values as weights. All ENM procedure was performed in the computational platform BIOENSEMBLES [30].

We converted the continuous suitability maps into binary predictions to represent species geographical ranges. We used thresholds that maximize sensitivity, defined when 95% of presences were correctly predicted [31,32]. This method is recommended when true absence data are lacking and to avoid overly broad predictions [33]. Thresholds were used to define species range size in both current and LGM scenarios. We calculated each species’ range as the sum of their total suitable cells in both present and LGM (Fig A in S1 File). ΔRange was then defined as a proportion to allow for comparisons between different range sizes, as follows:

$$\Delta Range=\frac{Present\;Range-LGM\;Range}{LGM\;Range}.$$ (1)

We also analyzed differences in suitability values as a proxy to species' abundance [34,35]. Based on the assumption that population dynamics are in equilibrium with the environment, high suitability indicates areas that best correspond to species' ecological niche. Therefore, areas of high environmental suitability are expected to be areas of high abundance as well [34]. Suitability ranges from zero (low suitability) to one (high suitability), thus very low suitability values (e.g. 0.03) are considered as background noise. We reduced background noise from cells with low suitability by removing cells with suitability below the established threshold in either present-day or LGM predictions. This method reduces commission errors, because it removes unsuitable habitat from the range maps. Suitable cells in either time period were maintained in all periods to better assess differences between the two scenarios. We computed the mean suitability in the present (S_PRE_) and LGM (S_LGM_) by summing all suitable cell values and dividing the total by the number of cells of occurrence for each species. Later ΔSuitability was also defined as a proportion:

$$\Delta Suitability=\frac{S_{PRE}-S_{LGM}}{S_{LGM}}.$$ (2)

### Statistical analysis

We used IUCN’s categories of threat as a proxy to extinction risk in extant mammals [36,37], treating it as a binomial response where CR, EN and VU are “threatened” and NT and LC are “not threatened” [38]. For these analyses DD species were removed, leaving 4183 species. We built General Linear Mixed-Effects Models (GLMMs) to test the extent with which the variation in geographic range (ΔRange) and abundance (ΔSuitability) between the present-day and LGM predict current mammals extinction risk. GLMM properly deals with nonnormal data (such as our binary threat status) by using binary family responses and incorporating random effects [39].

Because body size is often correlated with mammals extinction risk, we included body mass as a variable to account for random effects in all our GLMMs to avoid biased results [38,40]. Body mass data was obtained from the dataset available in [41], which is largely based on the PanTHERIA dataset [42]. ΔRange, ΔSuitability and body mass were log-transformed. Later we converted body mass into a categorical variable by rounding the log-transformed values. This process generated 15 distinct categories, ranging from the smallest (weighting between 1.77 and 4.44g) to the largest mammals (2285.94 to 3824.54kg).

Species’ geographic range size and reduction are considered in IUCN’s assessments of extinction risk, following criterion B [18]. Thus, it is expected that current range size is correlated to IUCN's threat status. Any eventual correlation found between the variation in geographic range (ΔRange) and threat status could be reflecting this relationship. We calculated Pearson’s correlation between the present-day range and our untransformed ΔRange (r = 0.06) and log-transformed ΔRange (r = 0.04). Given these low correlation coefficients, we feel confident our results are not a mere artifact of the potential relationships mentioned above. Furthermore, we analyze deep historical time through ENM, thus contractions or expansions found are influenced solely by differences in climatic variables between the present-day and the LGM. This is not a procedure used by IUCN, since their historical contractions are usually of the last 500 years and accounts mainly for anthropic actions.

We also tested ΔRange and ΔSuitability as predictors of population trend, which is available in IUCN red list. Population trend was treated as a binomial response variable. We unified the categories “stable” and “increasing”, which opposed to “decreasing” populations. DD species were kept but species with unkown population trends were removed, remaining 2729 species.

Similarly, we used chi-squared contingency table analysis to compare if differences in response to climate change in threatened and non-threatened species differ from expected by chance [43]. We converted both predictor variables ΔRange and ΔSuitability into categories by considering the frequencies of range contraction or population decline (negative values) versus range expansion or population increase (positive values). Finally, we used student t-tests to compare if responses significantly differ for these two groups.

To assess variation in species suitability between the transition Pleistocene-Holocene we mapped (1) biotic stability, number of cells where species occur in both the LGM and present-day (component A), (2) biotic gain, number of cells where species were not present in the LGM but are in present-day, (3) biotic loss, number of cells where species are present in the LGM but not in present-day (component B), and (4) the proportional loss of species, given by:

$$\mathrm{Proportional}\;\mathrm{loss}=\frac{B}{A+B}.$$ (3)

Because diversity is not randomly distributed at broad geographic scales [44] we also followed a deconstruction of biodiversity patterns approach [45]. This method decomposes richness into distinguished groups of species to better assess large-scale patterns [46], and we applied it here to better assess extinction risk in different mammal groups and scales. Our criteria were: (1) groups (hereafter 'taxa') with phylogenetic relationships among species at levels lower than class and higher than genus and (2) taxa occurring in the same biogeographic realm. Biogeographic realms were defined as Afrotropic, Australasia, Indomalaya, Nearctic, Neotropic and Palearctic [47]. Our approach resulted in nine monophyletic taxa, one paraphyletic taxon (Ungulata) and one group of minor clades composed of orders with less than 20 species (Table C in S1 File; [15]). The order Perissodactyla only has 15 species, thus we decided to create the paraphyletic group Ungulata (Perissodactyla + Artiodactyla) instead of adding Perissodactyla to the Minor Clades. This allows for comparisons with previous works that used Ungulata as a group (see [15,36,38]). In realms where an order does not occur, taxa are characterized only by extant orders present (e.g. Pholidota does not occur in the Neotropic, only Carnivora). Furthermore, taxa with less than 20 species occurring in a realm were excluded from analyses.

All statistical analyses were carried out in R version 3.4.0. GLMMs were built using the package "lme4" [48] using a binomial distribution, logit link and bobyqa optimizer. We also calculated coefficients of determination in the form of marginal R^2^, the variance explained by the fixed effects only, and conditional R^2^, variance explained by fixed and random effects combined [49], for all GLMMs using the rsquared function in the package "piecewiseSEM" [50].

## Results

**Fig 1:**
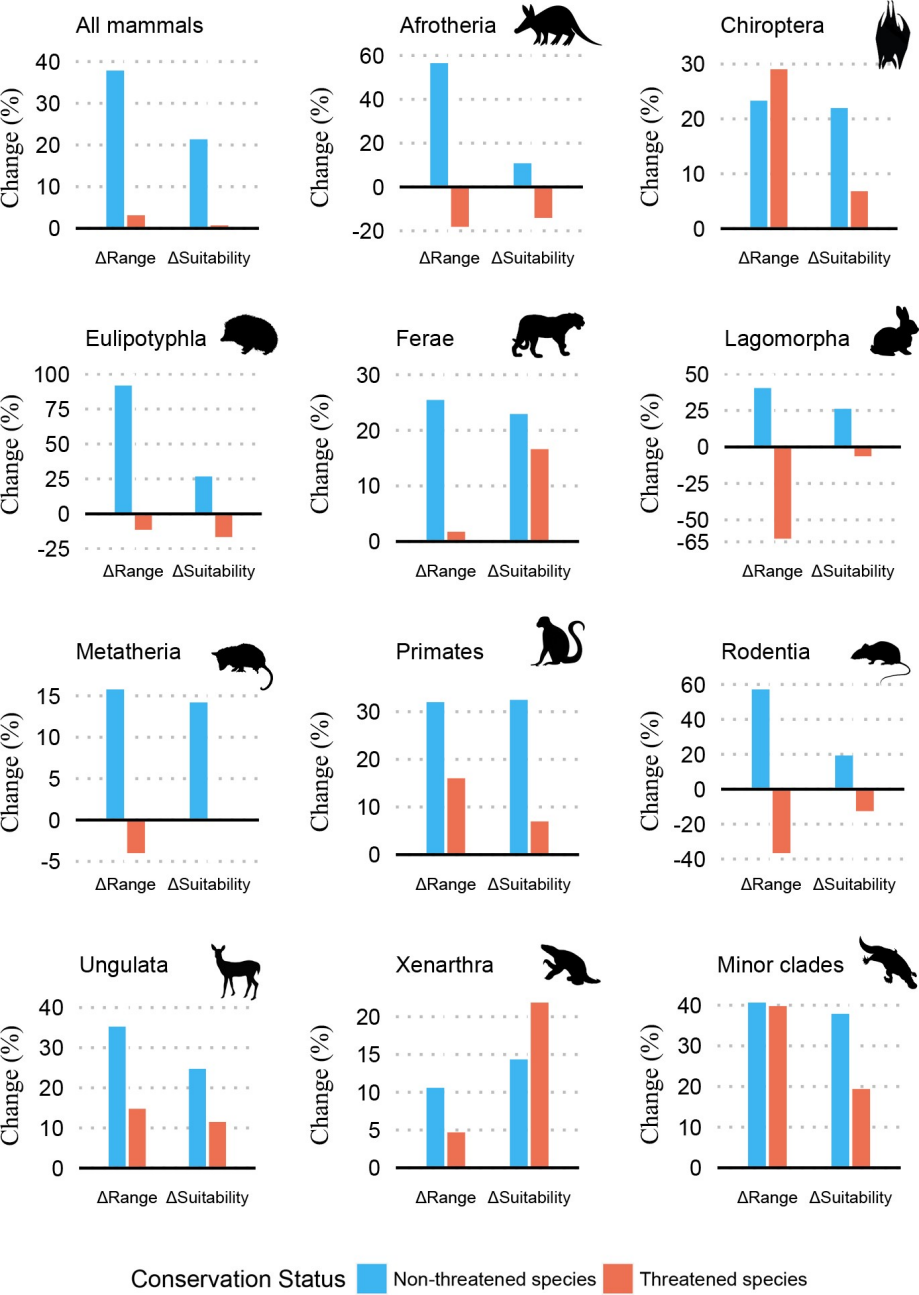
Change (%) in range size (ΔRange) and suitability (ΔSuitability) between the last glacial maximum and present-day. Results are for all non-threatened and threatened species, for different taxa and minor clades. See Table D in S1 File for raw values in sample size, range size, suitability and mean variation.

Threatened mammals (n = 749) presented stronger range contraction than non-threatened mammals (n = 3390) between the LGM and present-day (Fig 1; Table D in S1 File). Overall, 47% of threatened species underwent range contractions compared to only 24.1% of non-threatened species. Similarly, 53% of threatened species had range expansions against 75.9% of non-threatened species (t = 13.409; df = 1004.2; p<0.001). Mean suitability decreased for 46% of threatened species compared to only 20.8% of non-threatened species, and increased for 70.2% of non-threatened and 54% of threatened species (t = 14.873; df = 1154.5; p<0.001). Amongst non-threatened species Eulipotyphla presented the greatest range expansion (92.9%), and Lagomorpha had the greatest range contraction amongst threatened species (-63.9%). Suitability increased for all non-threatened species and decreases occurred only for threatened Afrotheria, Eulipotyphla, Lagomorpha and Rodentia (Fig 1).

Range contraction and decline in suitability alone explained 11.3% and 13.8% of mammal's extinction risk, respectively, and 36% and 39% when combined with body size (Table 1). Extinction risks for Afrotheria, Eulipotyphla, Lagomorpha, Rodentia and Primates were better explained by both range and suitability dynamics through the last ice age than for Chiroptera, Metatheria, and Ungulata. Extinction risks for Ferae, Xenarthra and minor clades were not significantly related to range and suitability dynamics (Table 1).

**Table 1:** Results of general linear mixed-effects models for our global models testing if range size change (ΔRange) and suitability change (ΔSuitability) between the last glacial maximum and present-day predict species’ threatened status.

		ΔRange				ΔSuitability			
Taxa	N	Estimate	SE	M.R^2^	C.R^2^	Estimate	SE	M.R^2^	C.R^2^
All species	4179	-0.65***	0.04	0.113	0.36	-2.42***	0.16	0.138	0.39
Afrotheria	64	-2.99**	1.15	0.729	0.771	-8.99***	2.42	0.491	0.491
Chiroptera	875	-0.45***	0.12	0.04	0.204	-2.06***	0.43	0.08	0.231
Eulipotyphla	322	-1.42***	0.22	0.57	0.57	-5.04***	0.86	0.596	0.596
Ferae	245	0.04	0.18	0	0.155	-0.55	0.51	0.009	0.149
Lagomorpha	77	-1.02*	0.41	0.297	0.347	-5.45**	1.96	0.558	0.618
Metatheria	283	-0.49**	0.16	0.052	0.255	-1.66**	0.54	0.053	0.259
Primates	353	-0.52***	0.10	0.107	0.181	-2.47***	0.41	0.161	0.255
Rodentia	1689	-0.91***	0.07	0.29	0.331	-3.46***	0.30	0.343	0.388
Ungulata	225	-0.12	0.13	0.005	0.005	-1.50**	0.49	0.062	0.062
Xenarthra	25	-0.53	1.49	0.019	0.019	1.68	3.37	0.024	0.024
Minor Clades	21	-0.79	8.16	0	0.99	-1.84	17.7	0	0.99

N = number of species. Estimate is the direction of the response; negative values represent range contractions and reduced suitability, positive values represent range expansions and increased suitability, for ΔRange and ΔSuitability respectively. SE = Standard Error. M. R^2^ = Marginal R^2^, it is R^2^ based on the fixed effects (ΔRange or ΔSuitability). C. R^2^ = Conditional R^2^, it is R^2^ based on both fixed and random effects (body size). Significance levels are indicated by asterisks:

* p < 0.05

** p < 0.01

*** p < 0.001.

**Fig 2:**
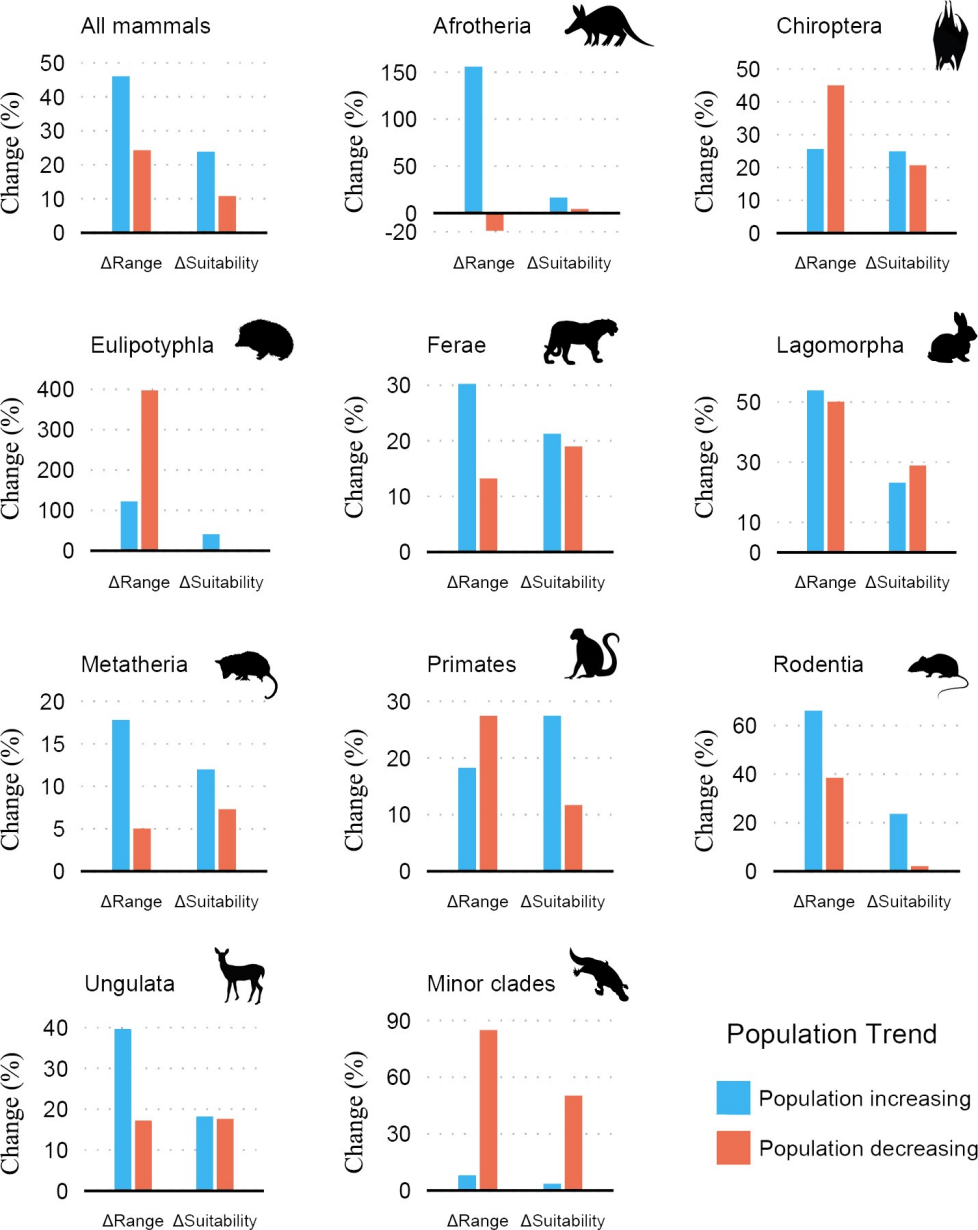
Change (%) in range size (ΔRange) and suitability (ΔSuitability) between the last glacial maximum and present-day. Results are for all species with increasing or decreasing population trends, for different taxa and minor clades. See Table E in S1 File for raw values in sample size, range size, suitability and mean variation.

Change patterns in population trends for increasing (n = 1366) and decreasing (n = 1360) population showed range expansions and increased suitability (Fig 2; Table E in S1 File). Eulipotyphla, Primates and Rodentia were the only taxa with a significant association between both range contraction and suitability decline with decreasing population (Table 2). In models for Eulipotyphla, ΔRange and ΔSuitability had a stronger explaining power (R^2^ = 35%), while models for Primates and Rodentia all presented Marginal R^2^'s lower than 10%. Results for conservation status and population trend were similar as from chi-square tests (Tables F-G in S1 File) as for biogeographical realms (see Text A; Figs B-G, Tables H-I in S1 File).

**Table 2:** Results of general linear mixed-effects models for our global models testing if range size change (ΔRange) and suitability change (ΔSuitability) between last glacial maximum and present predict species’ current population trends. Xenarthra was not included in analysis because only 13 species had a known population trend.

		ΔRange				ΔSuitability			
Taxa	N	Estimate	SE	M. R^2^	C. R^2^	Estimate	SE	M. R^2^	C. R^2^
All species	2726	-0.26***	0.04	0.026	0.133	-1.09***	0.12	0.044	0.152
Afrotheria	36	-1.19	0.73	0.209	0.280	-4.84	3.25	0.179	0.249
Chiroptera	450	0.14	0.11	0.004	0.104	-0.30	0.33	0.002	0.089
Eulipotyphla	157	-0.45***	0.12	0.15	0.15	-2.44***	0.49	0.346	0.346
Ferae	196	-0.04	0.17	0.000	0.000	-0.34	0.43	0.004	0.004
Lagomorpha	40	-0.13	0.26	0.008	0.008	0.43	0.71	0.012	0.012
Metatheria	207	0.01	0.16	0.000	0.118	-0.49	0.53	0.005	0.107
Primates	298	-0.35*	0.17	0.041	0.227	-1.71**	0.62	0.072	0.268
Rodentia	1102	-0.45***	0.06	0.088	0.177	-1.55***	0.20	0.102	0.198
Ungulata	205	0.12	0.15	0.005	0.015	-0.38	0.49	0.004	0.019
Minor Clades	22	0.97	0.80	0.177	0.177	9.46	4.82	0.740	0.704

N = number of species. Estimate is the direction of the response; negative values represent range contractions and reduced suitability, positive values represent range expansions and increased suitability, for ΔRange and ΔSuitability respectively. SE = Standard Error. M. R^2^ = Marginal R^2^, it is R^2^ based on the fixed effects (ΔRange or ΔSuitability). C. R^2^ = Conditional R^2^, it is R^2^ based on both fixed and random effects (body size). Significance levels are indicated by asterisks:

* p < 0.05

** p < 0.01

*** p < 0.001.

**Fig 3:**
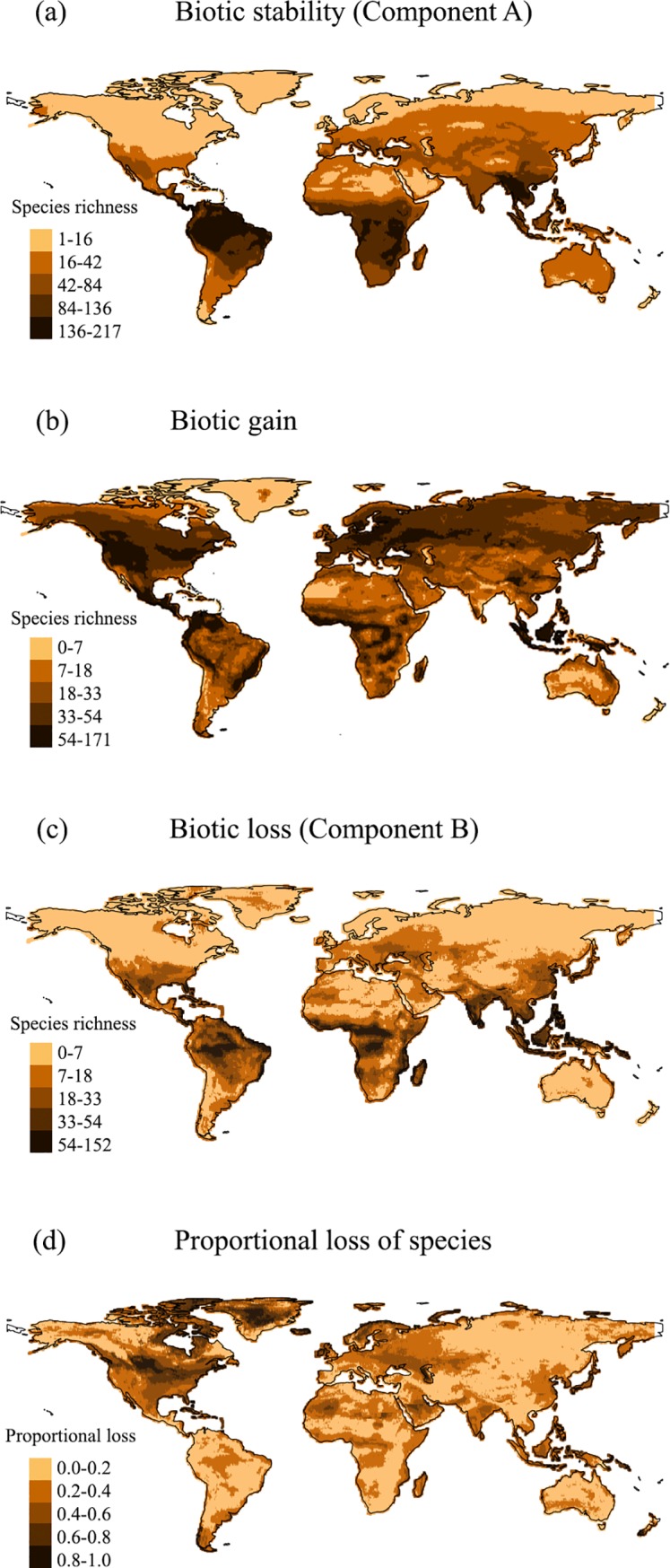
Variation in species suitability between the transition Pleistocene-Holocene. (a) Biotic stability (Component A), cells where mammals occur in both present-day and LGM. (b) Biotic gain, cells where mammals did not occur in the LGM but occur in present-day. (c) Biotic loss, cells where mammals were present in the LGM but lost in present-day. (d) Proportional loss of mammals, calculated by B/A+B. Maps made with Natural Earth.

Stable areas where species persisted in both present-day and LGM are all located in the tropics (Fig 3A). Most species gained suitable habitats continuously across the Palearctic and Nearctic, and in a disjunctive manner in Indonesia, the Neotropic (Central America and along the northwest and east coasts of South America) and Afrotropic (west equatorial Africa) (Fig 3B). Species lost suitable habitats across the tropical regions over the last ice age, especially in the western Amazon basin, west equatorial Africa and Southeast Asia (Fig 3C). However, when combined with stable habitats, Nearctic presented the greatest proportional loss of species, together with Palearctic and Indomalaya realms (Fig 3D).

## Discussion

Our results from ecological niche modeling, the standard conceptual and analytical tool to address climate change in a macroecological perspective, support our predictions and show that Quaternary dynamics of climatic suitability and species geographical ranges are correlated to the current mammal’s extinction risk. Species that benefited from climate change with large range expansions and population increases since the LGM are currently less prone to extinction (non-threatened mammals) than species showing range contractions and population decreases to some extent (threatened mammals).

Our findings demonstrate range contractions and abundance declines for mammals at wide spatial and temporal scales. Most importantly, our findings establish historical range and habitat suitability variation through time as relevant predictors to extinction risk for studies in the future. At global and biogeographical scales, all groups that underwent significant Quaternary range contractions have currently more species listed in IUCN’s threat categories than groups whose ranges did not contract significantly. We believe this reinforces the strength of using range contractions as a key predictor of extinction risk into climate change scenarios. Complementarily, studies at local scales have also registered abundance decline and/or range contractions in small mammals (but specially in rodents) between the LGM and present-day in the Pacific coast of the United States [51,52], Mexico [53] and Morocco [54]. Recent anthropogenic climate change has also been appointed as the cause of extinction of the rodent *Melomys rubicola* in northern Australia [55] and declines of rodent diversity in Spain [56].

Additionally, our findings show that species loss is not randomly distributed across the globe, as most mammals losing geographical range since the LGM occur mainly in the Southern hemisphere. Past Quaternary climate oscillations were most evident as changes in temperature at higher latitudes, allowing for range expansions as the landscape became ice-free and was rapidly recolonized [57]. These temperature-dependent dynamics are probably more important at high latitudes and may be responsible for the regional extinctions of small-ranged species during glaciations [58]. On the other hand, Quaternary climate oscillations in the tropics had larger shifts in precipitation than in temperature, this possibly resulted in more range contractions than expansions, since no ice-free landscape became available [57]. This greater climatic stability of tropical latitudes may have allowed for greater persistence of species and few extinctions during glaciations, even if in smaller ranges and saturated niches [58].

These areas are also congruent with a more recent study where predicted hotspots of future climate change risk for mammals are concentrated in Papua-New Guinea, central and eastern Sub-Saharan Africa and the western portion of the Amazon river between Peru and Ecuador [59]. Tropical terrestrial species are expected to be more sensitive to climate change because of its often-restricted physiological tolerances [60,61]. Moreover, areas of climatic instability, with consequent species' range contractions and limited opportunity for tracking suitable habitats, are located mainly in the Tropics, Greenland and parts of Australia [11], also equivalent to areas we found of lost suitability from the LGM to the present-day.

Predictive power of range contraction and abundance decline were particularly high for threatened species in notoriously small-bodied orders like Rodentia, Eulipotyphla and Lagomorpha. In Afrotheria, a taxon where range contraction had a predictive power of 71%, 11 out the 14 threatened species belong to the order Afrosoricida, likewise composed of small mammals such as golden-moles and tenrecs [62]. Small-bodied species usually have low dispersion abilities and consequently have more difficulty in tracking climate changes [63]. Metatheria, Chiroptera and Primates are generally good dispersers, thus life-history traits (e.g. reproduction age, litter size, fertility rate) are usually factors more important when assessing their extinction risk [64–66], which could explain range and suitability change's low predictive power in these groups.

It is expected from communities that faced challenges in the past to be more resilient to them in the present [67]. Hence absence of responses in our models for groups such as ungulates, Carnivores and Xenarthra, may be due to most species in these taxa being already extinct (see [68]). Species more sensitive to Quaternary climate change did not survive into modern times and were not included in our models. As such, we did not capture the effects of Quaternary climate change on current ungulates, Carnivores and Xenarthra. Nonetheless, species belonging to these taxa are still threatened by future climate change and general environmental variation [12,69,70] and more investigation incorporating fossil records from the Quaternary period may be needed in this aspect.

Most mammals are commonly threatened by habitat loss, modification and fragmentation [71,72]. Such threat coupled with negative response to past climate change imply a worrisome future for mammals in rapidly changing human-induced climates, especially because extinction is a synergetic process influenced by the interaction of multiple threats [73]. Inclusion of historical records has increased the precision of models predicting future threats [74,75], improves our understanding of extant mammalian trait turnover [70] and may help analyze species vulnerability to future anthropogenic impacts. Thus, fossil record, past climate change and historical range dynamics become important variables to be incorporated into future extinction risk analyses and conservation planning.

## Supporting information

S1 File Document containing further results, multiple tables and figures. (PDF)
