# Supplementary material for: Historical range contractions can predict extinction risk in extant mammals
Source: PLoS One. 2019 Sep 5;14(9):e0221439. doi: 10.1371/journal.pone.0221439 (PMC6728145; doi:10.1371/journal.pone.0221439)
Supplement: S1 File — (PDF) [file pone.0221439.s001.pdf]

S1 FILE

SUPPORTING INFORMATION

Text A. Results for biogeographical realms

In all our biogeographical realms models, response to climate change was significantly different for non-threatened and threatened mammals (Table G), with non-threatened species benefiting more from climate change (Figs B-G). The only exception is Indomalaya, where response was range contraction for both categories (Fig D). Areas where species were present in the LGM but lost in the present-day are located mainly in the tropics.

In all realms range contraction and suitability decline significantly explain mammals' extinction risk (Table H). However, with the exception of the Nearctic, body size explained most of the threatened status. In models for population trend, variables explaining power were low and only significant for the Afrotropic, Nearctic and the Neotropic (Table I).

Our models for Rodentia were systematically significant globally and in all realms except in the Indomalaya. Eulipotyphla and Primates were the other taxa with the most significant responses for both ΔRange and ΔSuitability as predictors of extinction risk and population decline. Body size was overall not an important predictor of extinction risk and population decline for Rodentia and Eulipotyphla, but it was for Primates, explaining alone more than 20% of the extinction risk in the Afrotropic and Indomalaya.

SUPPLEMENTARY TABLES

Table A. Number of species listed by biogeographic realms and conservation categories. Total number of extant mammals taken from the The IUCN Red List of Threatened Species. Number of species are listed by realms (some may occur in more than one) and conservation categories.

Realm	All species	Non-threatened	Threatened	DD
World	4645	3390	793	462
Afrotropic	1133	824	203	106
Australasia	627	446	128	53
Indomalaya	888	624	186	78
Nearctic	513	474	30	9
Neotropic	1425	1052	203	170
Palearctic	905	729	108	68

Table B. Principal climatic variables, in order of importance, selected by Factor Analysis for each biogeographic realm and used in Ecological Niche Modeling.

Biogeographic realm	Climate layers
Afrotropic	Precipitation of Wettest Quarter
	Precipitation of Driest Quarter
	Mean Temperature of Warmest Quarter
	Mean Diurnal Range
	Mean Temperature of Coldest Quarter
Australasia	Precipitation of Wettest Quarter
	Precipitation of Driest Quarter
	Mean Temperature of Warmest Quarter
	Mean Diurnal Range
Indomalaya	Precipitation of Wettest Quarter,
	Precipitation of Driest Quarter,
	Annual Mean Temperature,
	Temperature Seasonality
Nearctic	Precipitation of Wettest Month
	Max Temperature of Warmest Month
	Temperature Annual Range
	Precipitation Seasonality
Neotropic	Precipitation of Wettest Quarter
	Precipitation of Driest Quarter
	Annual Mean Temperature
	Mean Diurnal Range
	Isothermality
Palearctic	Temperature Seasonality
	Precipitation of Wettest Month
	Precipitation of Driest Month
	Max Temperature of Warmest Month
	Mean Diurnal Range

Table C. Species' information. Monophyletic and paraphyletic taxa used in analysis and their respective composing taxa, species' common names, and number of non-threatened and threatened species. The orders Dermoptera, Scandentia and Monotremata were grouped as "Minor clades" because of low species number independently.

Taxa	Orders	Common names	Non-threatened Species	Threatened species
Afrotheria	Afrosoricida	Golden moles and tenrecs	33	11
	Hyracoidea	Hyraxes	5	0
	Macroscelidea	Elephant shrews	11	1
	Proboscidea	Elephants	0	2
	Tubulidentata	Aardvark	1	0
Chiroptera	-	Bats	769	106
Eulipotyphla	-	Hedgehogs, moles, and shrews	286	36
Ferae	Carnivora	Cats, bears, raccoons	177	60
	Pholidota	Pangolins	0	8
Lagomorpha	-	Rabbits, hares and pikas	65	12
Metatheria	Didelphimorphia	Opossums	74	4
	Diprotodontia	Kangaroos, wallabies, koalas, wombats	83	35
	Dasyuromorphia	Carnivorous marsupials	56	10
	Notoryctemorphia	Marsupial moles	2	0
	Microbiotheria	Monito del monte	0	1
	Paucituberculata	Shrew opossums	4	1
	Peramelemorphia	Bandicoots and bilbies	9	4
Primates	-	Monkeys and apes	143	210
Rodentia	-	Mice, squirrels, hamsters, capybaras	1505	184
Ungulata	Cetartiodactyla	Deer, alpacas, giraffes, antelopes and camels	121	89
	Perissodactyla	Horses, zebras, tapirs and rhinoceroses	4	11
Xenarthra	Cingulata	Armadillos	14	2
	Pilosa	Anteaters and sloths	7	2
Minor Clades	Dermoptera	Flying lemurs	2	0
	Scandentia	Treeshrews	15	0
	Monotremata	Platypus and echidnas	2	2

Table D. Raw values for conservation status. Number of non-threatened and threatened species according to The IUCN Red List of Threatened Species. Taxa are listed with their total range size, mean suitability and historical differences combined for the last glacial maximum and the present-day.

	Non-threatened species							Threatened species						
		Range			Suitability				Range			Suitability		
Groups	N	LGM	PRE	ΔR (%)	LGM	PRE	ΔS (%)	N	LGM	PRE	ΔR (%)	LGM	PRE	ΔS (%)
Afrotheria	50	30816	48450	57.2	0.77	0.86	11.5	14	7945	6445	-18.9	0.83	0.71	-14.8
Chiroptera	769	963677	1191126	23.6	0.66	0.81	22.3	106	10708	13849	29.3	0.74	0.79	7.1
Eulipotyphla	286	169425	326865	92.9	0.61	0.79	27.9	36	2368	2072	-12.5	0.83	0.68	-17.7
Ferae	177	566994	712959	25.7	0.66	0.81	23.2	68	106584	108744	2.03	0.67	0.78	16.9
Lagomorpha	65	75295	106485	41.4	0.65	0.82	27.2	12	1692	611	-63.9	0.74	0.69	-7.42
Metatheria	229	93129	107994	15.9	0.69	0.79	14.4	54	6668	6389	-4.18	0.74	0.74	0.23
Primates	143	65405	86550	32.3	0.63	0.83	32.8	210	23483	27323	16.4	0.72	0.77	7.29
Rodentia	1505	764274	1207956	58.1	0.67	0.81	20.2	184	25229	15772	-37.5	0.79	0.68	-13.4
Ungulata	125	183109	248353	35.6	0.64	0.80	25.1	100	83140	95745	15.2	0.70	0.78	11.9
Xenarthra	21	42937	47569	10.8	0.62	0.71	14.6	4	9949	10436	4.89	0.56	0.68	22.1
Minor Clades	19	7249	10223	41.0	0.52	0.72	38.3	2	249	349	40.2	0.63	0.75	19.8

Non-threatened species = species listed as Least Concerned (LC) or Near Threatened (NT) by IUCN. Threatened species = species listed as Vulnerable (VU), Endangered (EN) and Critically Endangered (CR) by IUCN. Data deficient (DD) species were excluded from analysis. N = species number. Range = number of cells occupied. Suitability = mean suitability of total probable range. ΔR (%) = the difference in range size between the LGM and present (PRE). ΔS (%) = the difference in adequate suitability between the LGM and present.

Table E. Raw values for population trends. Number of species with increasing or decreasing population trends according to The IUCN Red List of Threatened Species. Taxa are listed with their total range size, mean suitability and historical differences combined for the last glacial maximum and present-day.

	Population increasing							Population decreasing						
		Range			Suitability				Range			Suitability		
Groups	N	LGM	PRE	ΔR (%)	LGM	PRE	ΔS (%)	N	LGM	PRE	ΔR (%)	LGM	PRE	ΔS (%)
Afrotheria	9	12084	31156	157.8	0.70	0.82	18.37	27	7665	6067	-20.9	0.80	0.85	6.38
Chiroptera	237	356618	450038	26.20	0.66	0.83	25.48	213	84764	123425	45.61	0.67	0.81	21.26
Eulipotyphla	94	71094	161290	126.9	0.55	0.80	45.40	63	4173	20943	401.9	0.74	0.71	-4.06
Ferae	73	311513	406724	30.56	0.66	0.81	21.60	123	278333	316106	13.57	0.67	0.80	19.31
Lagomorpha	17	18468	28526	54.46	0.66	0.82	23.76	23	17192	25926	50.80	0.62	0.80	29.52
Metatheria	94	50844	60004	18.02	0.70	0.79	12.21	113	28384	29874	5.25	0.71	0.77	7.52
Primates	38	27056	32081	18.57	0.65	0.83	27.77	260	48567	62056	27.77	0.70	0.79	12.04
Rodentia	732	418988	699224	66.88	0.66	0.82	24.40	370	93095	129654	39.27	0.73	0.75	2.90
Ungulata	62	92568	129665	40.08	0.65	0.77	18.64	143	156588	184263	17.67	0.68	0.80	18.09
Minor Clades	5	4252	4636	9.03	0.74	0.78	4.59	17	3277	6094	85.96	0.46	0.70	51.29

Data deficient (DD) species were included in analysis. N = species number. Range = number of cells occupied. Suitability = mean suitability of total probable range. ΔR (%) = the difference in range size between the LGM and present (PRE). ΔS (%) = the difference in adequate suitability between the LGM and present.

Table F. Results of chi-square test for independence to determine if Conservation Status (threatened or non-threatened) and Population Trend (increasing or decreasing) are related to range contraction or expansion (ΔRange) and suitability increase or decrease (ΔSuitability) in our global models. Xenarthra was not included in the Population Trend analysis because only 13 species had a known population trend.

	Conservation Status					Population Trend				
		ΔRange		ΔSuitability			ΔRange		ΔSuitability	
Taxa	N	χ^2	df	χ^2	df	N	χ^2	df	χ^2	df
All species	4179	166.21***	1	212.31***	1	2726	61.312***	1	76.815***	1
Afrotheria	64	17.627***	1	16.384***	1	36	0.857	1	0.184	1
Chiroptera	875	5.278***	1	21.257***	1	450	2.648	1	8.488**	1
Eulipotyphla	322	47.177***	1	42.811***	1	157	21.967***	1	25.272***	1
Ferae	245	0.459	1	0.0134	1	196	0.099	1	0.000002	1
Lagomorpha	77	6.442***	1	14.873***	1	40	2.764	1	1.954	1
Metatheria	283	5.459**	1	9.269**	1	207	0.133	1	1.506	1
Primates	353	28.65***	1	46.011***	1	298	87.678***	1	3.164	1
Rodentia	1689	154.61***	1	160.25***	1	1102	87.678***	1	95.645***	1
Ungulata	225	0.539	1	1.532	1	205	1.363	1	2.591	1
Xenarthra	25	0.446	1	0.074	1	13	-	-	-	-
Minor Clades	21	0.233	1	0.1105	1	22	7.48**	1	3.562	1

Data deficient species were excluded from analysis for Conservation Status and included for Population Trend. N = total species number. χ^2 = Chi-square value. df = degrees of freedom. Significance levels are indicated by asterisks: * $p < 0.05$; ** $p < 0.01$; *** $p < 0.001$.

Table G. Results of chi-square test for independence to determine if Conservation Status (threatened or non-threatened) and Population Trend (increasing or decreasing) are related to range contraction or expansion (ΔRange) and suitability increase or decrease (ΔSuitability) in our biogeographical realms models.

	Conservation Status					Population Trend				
		ΔRange		ΔSuitability			ΔRange		ΔSuitability	
Realm	N	χ^2	df	χ^2	df	N	χ^2	df	χ^2	df
Afrotropic	1027	37.6***	1	63.172***	1	617	1.0374	1	3.959*	1
Australasia	574	28.469***	1	42.458***	1	396	2.079	1	5.314*	1
Indomalaya	810	0.092	1	2.005	1	532	2.3955	1	0.248	1
Nearctic	504	53.353***	1	40.596***	1	387	27.166***	1	29.568***	1
Neotropic	1255	28.737***	1	47.633***	1	850	24.348***	1	35.701***	1
Palearctic	837	17.3***	1	21.417***	1	463	10.418**	1	12.074***	1

Data deficient species were excluded from analysis for Conservation Status and included in Population Trend. N = total species number. χ^2 = Chi-square value. df = degrees of freedom. Significance levels are indicated by asterisks: * $p < 0.05$; ** $p < 0.01$; *** $p < 0.001$.

Table H. Results of general linear mixed-effects models for our biogeographical realms models testing if ΔRange and ΔSuitability predict species' threatened status. Taxa with fewer than 20 species occurring in a realm are not shown.

		ΔRange				ΔSuitability			
Realm	Groups	Estimate	SE	M. R^2	C. R^2	Estimate	SE	M. R^2	C. R^2
Afrotropic	All species	-0.64***	0.08	0.104	0.382	-3.18***	0.37	0.128	0.433
	Afrotheria	-2.68***	0.81	0.710	0.710	-8.87***	2.47	0.474	0.474
	Chiroptera	-0.08	0.35	0.002	0.002	-1.73	1.22	0.055	0.055
	Eulipotyphla	-1.95***	0.43	0.661	0.661	-6.76***	1.50	0.572	0.572
	Ferae	0.101	0.25	0.003	0.013	-0.15	1.09	0	0
	Primates	-0.61***	0.17	0.134	0.338	-3.64***	0.85	0.213	0.430
	Rodentia	-1.15***	0.23	0.326	0.497	-4.98***	1.05	0.349	0.504
	Ungulata	0.38	0.29	0.047	0.086	-0.54	0.86	0.006	0.006
Australasia	All species	-0.59***	0.11	0.066	0.352	-1.80***	0.39	0.05	0.355
	Chiroptera	-0.37	0.25	0.020	0.274	-2.24*	0.94	0.052	0.306
	Metatheria	-0.46**	0.17	0.051	0.272	-1.51**	0.55	0.05	0.273
	Rodentia	-1.09***	0.23	0.275	0.275	-3.04***	0.81	0.176	0.176
Indomalaya	All species	-0.19*	0.09	0.008	0.347	-0.53.	0.29	0.006	0.346
	Chiroptera	-0.30	0.19	0.029	0.029	-1.26.	0.65	0.045	0.045
	Eulipotyphla	-0.71	0.55	0.130	0.284	-2.22	1.64	0.114	0.267
	Ferae	-0.33	0.36	0.016	0.203	0.27	0.98	0.001	0.245
	Primates	-0.50*	0.25	0.090	0.305	-1.29	0.79	0.067	0.296
	Rodentia	-0.18	0.17	0.012	0.058	-0.12	0.59	0	0.051
	Ungulata	0.29	0.27	0.025	0.082	-0.10	0.92	0	0.031
Nearctic	All species	-1.03***	0.18	0.275	0.345	-3.29***	0.64	0.401	0.532
	Chiroptera	-0.43	0.49	0.055	0.055	-3.24***	2.11	0.304	0.304
	Eulipotyphla	-0.43	0.96	0.047	0.350	-4.05	4.06	0.410	0.748
	Carnivora	0.11	0.65	0.002	0.280	0.10	1.36	0	0.267
	Lagomorpha	-1.27	0.96	0.569	0.569	-6.45	3.92	0.743	0.743
	Rodentia	-1.68***	0.31	0.539	0.545	-5.83***	1.23	0.743	0.759

Neotropic	All species	-0.71***	0.09	0.096	0.304	-2.74***	0.32	0.104	0.323
	Chiroptera	-0.79**	0.30	0.079	0.079	-2.73**	0.96	0.087	0.087
	Eulipotyphla	-1.78**	0.66	0.517	0.517	-5.64*	2.26	0.422	0.422
	Carnivora	0.95.	0.50	0.098	0.098	2.39	1.88	0.058	0.058
	Metatheria	-0.66	0.80	0.066	0.066	-2.52	2.35	0.088	0.088
	Primates	-0.37.	0.20	0.038	0.065	-1.84	0.67	0.085	0.126
	Rodentia	-1.045***	0.13	0.271	0.308	-4.39***	0.52	0.293	0.350
	Ungulata	1.367	0.84	0.175	0.175	2.62	2.62	0.059	0.059
	Xenarthra	-0.461	1.44	0.014	0.014	2.60	3.63	0.049	0.049
Palearctic	All species	-0.292***	0.08	0.040	0.419	-1.34***	0.36	0.053	0.425
	Chiroptera	-0.133	0.23	0.011	0.011	0.31	0.79	0.005	0.005
	Eulipotyphla	-0.154	0.37	0.021	0.021	0.35	1.34	0.006	0.006
	Ferae	0.013	0.18	0.000	0.495	-0.90	0.89	0.017	0.485
	Lagomorpha	-0.735	0.46	0.215	0.215	-4.79.	2.69	0.453	0.453
	Rodentia	-0.475***	0.13	0.163	0.212	-1.94**	0.75	0.168	0.228
	Ungulata	-0.317.	0.18	0.058	0.058	-1.84*	0.82	0.108	0.108

Estimate is the direction of the response; negative values represent range contractions for ΔRange and reduced suitability for ΔSuitability, positive values represent range expansions and increased suitability, for ΔRange and ΔSuitability respectively. SE = Standard Error. M. R^2 = Marginal R^2, it is R^2 based on the fixed effects (ΔRange or ΔSuitability). C. R^2 = Conditional R^2, it is R^2 based on both fixed and random effects (Body size). Significance levels are indicated by asterisks: . $p < 0.10$; * $p < 0.05$; ** $p < 0.01$; *** $p < 0.001$.

Table I. Results of general linear mixed-effects models for our biogeographical realms models testing if ΔRange and ΔSuitability predict species' current population trends. Taxa with fewer than 20 species occurring in a realm are not shown.

					ΔRange				ΔSuitability			
Realm	Groups	N	0	1	Estimate	SE	M.R^2	C. R^2	Estimate	SE	M.R^2	C. R^2
Afrotropic	All species	617	289	328	-0.201*	0.08	0.014	0.145	-0.949**	0.32	0.017	0.154
	Afrotheria	35	9	26	-0.701	0.63	0.082	0.185	-4.390	3.26	0.144	0.223
	Chiroptera	71	27	44	1.331**	0.46	0.263	0.320	2.398*	1.18	0.099	0.178
	Eulipotyphla	45	18	27	-0.890*	0.36	0.260	0.260	-4.805**	1.59	0.373	0.373
	Ferae	64	26	38	0.115	0.29	0.003	0.003	2.066	1.58	0.038	0.038
	Primates	120	23	97	-0.503*	0.25	0.079	0.288	-4.158**	1.33	0.210	0.472
	Rodentia	178	153	25	-0.840***	0.24	0.215	0.316	-3.93***	0.98	0.263	0.364
	Ungulata	97	31	66	0.850**	0.30	0.205	0.205	3.708**	1.13	0.217	0.217
Australasia	All species	396	189	207	-0.067	0.11	0.001	0.111	-0.061	0.39	0.000	0.114
	Chiroptera	125	66	99	0.061	0.25	0.001	0.103	-0.049	0.93	0.000	0.097
	Metatheria	147	64	83	0.174	0.18	0.007	0.210	0.150	0.58	0.001	0.192
	Rodentia	98	55	43	-0.615**	0.23	0.110	0.110	-1.018	0.74	0.025	0.025
Indomalaya	All species	532	198	334	0.214*	0.09	0.015	0.198	0.730*	0.29	0.015	0.200
	Chiroptera	117	50	67	0.208	0.15	0.022	0.023	0.543	0.54	0.011	0.011
	Ferae	75	26	49	0.000	0.32	0.000	0.018	0.905	0.95	0.018	0.051
	Primates	68	2	66	-0.743	0.59	0.219	0.219	-2.092	2.35	0.194	0.194
	Rodentia	168	96	72	0.167	0.14	0.009	0.169	0.335	0.52	0.003	0.167
	Ungulata	58	9	49	0.809.	0.43	0.162	0.162	0.336	1.40	0.002	0.002
Nearctic	All species	387	303	84	-0.41***	0.12	0.057	0.143	-1.69***	0.33	0.169	0.294
	Chiroptera	66	58	8	-0.200	0.36	0.012	0.045	-1.523	1.19	0.088	0.088
	Eulipotyphla	42	37	5	-1.035*	0.45	0.32	0.32	-3.977*	1.91	0.634	0.634
	Carnivora	37	20	17	0.591	0.42	0.08	0.08	-1.376	0.86	0.096	0.096
	Rodentia	209	165	44	-0.61***	0.16	0.133	0.133	-1.97***	0.46	0.266	0.288
Neotropic	All species	850	469	381	-0.50***	0.08	0.052	0.25	-1.86***	0.30	0.053	0.264

	Chiroptera	139	101	38	-0.460.	0.27	0.034	0.034	-1.427	0.93	0.026	0.026
	Eulipotyphla	33	20	13	-0.893*	0.44	0.195	0.195	-3.394*	1.68	0.195	0.195
	Carnivora	50	16	34	-0.214	0.45	0.006	0.006	0.345	1.59	0.001	0.001
	Metatheria	60	30	30	-0.748	0.47	0.073	0.113	-4.290*	1.74	0.174	0.218
	Primates	96	12	84	-0.234	0.35	0.011	0.202	-0.591	1.01	0.008	0.205
	Rodentia	431	274	157	-0.68***	0.11	0.138	0.179	-3.13***	0.46	0.173	0.218
	Ungulata	21	6	15	2.175	1.82	0.194	0.688	10.482	6.93	0.353	0.693
Palearctic	All species	463	255	208	-0.117.	0.06	0.011	0.124	-0.543*	0.24	0.016	0.124
	Chiroptera	62	34	28	0.030	0.23	0.000	0.000	-0.248	0.62	0.003	0.003
	Eulipotyphla	41	31	10	0.082	0.19	0.007	0.007	-1.157	0.87	0.076	0.076
	Ferae	73	32	41	0.145	0.16	0.014	0.158	0.113	0.70	0.001	0.130
	Rodentia	182	122	60	-0.198.	0.11	0.028	0.200	-0.324	0.37	0.006	0.187
	Ungulata	68	22	46	-0.009	0.17	0.000	0.000	-0.781	0.79	0.020	0.020

N is the number of species. 0 represents species' stable or increasing and 1 represents species with decreasing current population trend according to The IUCN Red List of Threatened Species. Estimate is the direction of the response; negative values represent range contractions and reduced suitability, positive values represent range expansions and increased suitability, for ΔRange and ΔSuitability respectively. SE = Standard Error. M. R^2 = Marginal R^2, it is R^2 based on the fixed effects (ΔRange or ΔSuitability). C. R^2 = Conditional R^2, it is R^2 based on both fixed and random effects (Body size). Significance levels are indicated by asterisks: . $p < 0.10$; * $p < 0.05$; ** $p < 0.01$; *** $p < 0.001$.

SUPPLEMENTARY FIGURES

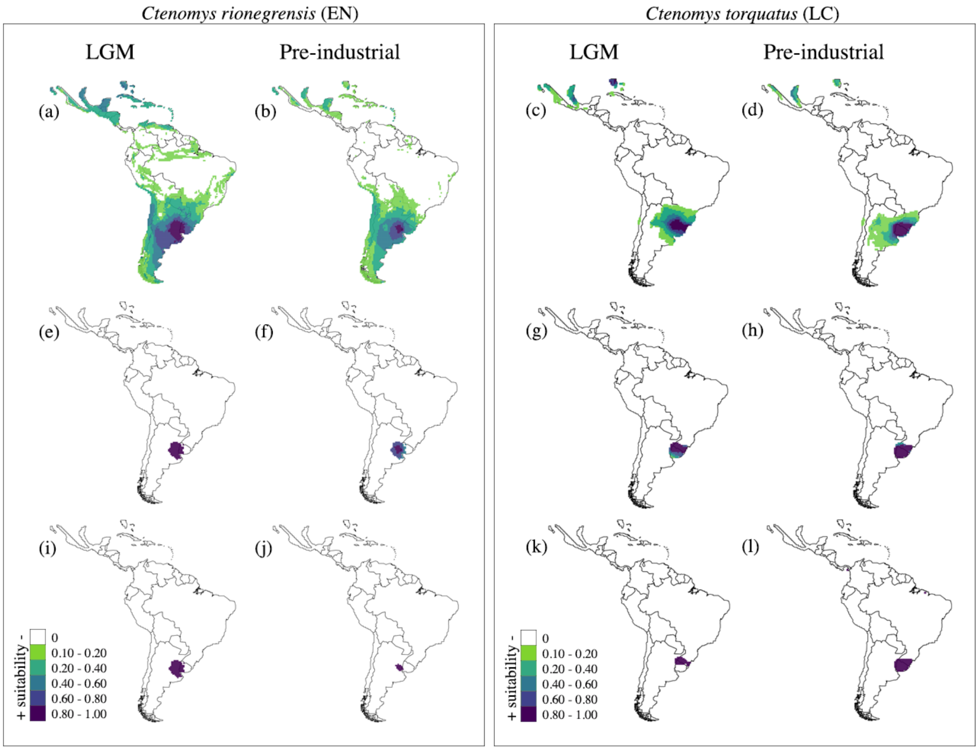

Fig A. Examples from the order Rodentia for two species in the same genus and different IUCN Red List of Threatened Species' categories. *Ctenomys rionegrensis*, listed as Endangered (EN), shows range contraction and reduced suitability between the LGM and present. *Ctenomys torquatus*, listed as Least Concern (LC), shows range expansion and increased suitability between the LGM and present. (a-d) Modeled suitability for the Last Glacial Maximum and present-day, respectively, for both *C. rionegrensis* and *C. torquatus.* (e-h) Continuous suitability maps. (i-l) Binary presence and absence maps. Suitability was cut above threshold value 0.81 for *C. rionegrensis* and 0.86 for *C. torquatus*, in both LGM and present.

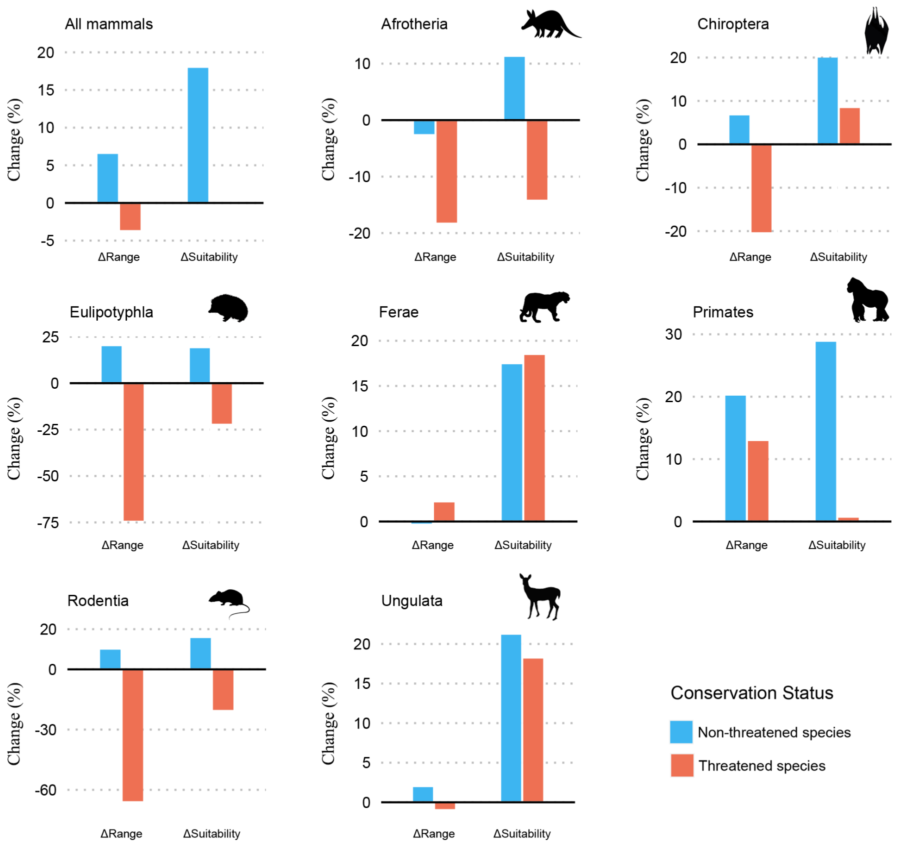

Fig B. Change (%) of range size (ΔRange) and suitability (ΔSuitability) between the last glacial maximum and the present-day, respectively, for all non-threatened and threatened species, for different taxa and minor clades occurring in the Afrotropic.

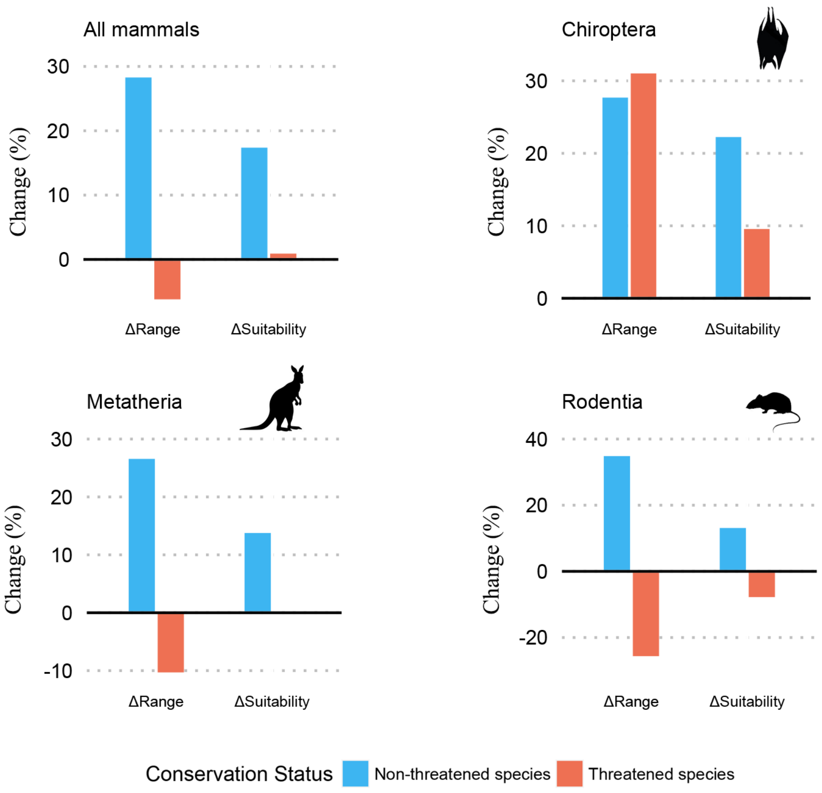

Fig C. Change (%) of range size (ΔRange) and suitability (ΔSuitability) between the last glacial maximum and the present-day, respectively, for all non-threatened and threatened species, for different taxa and minor clades occurring in Australasia.

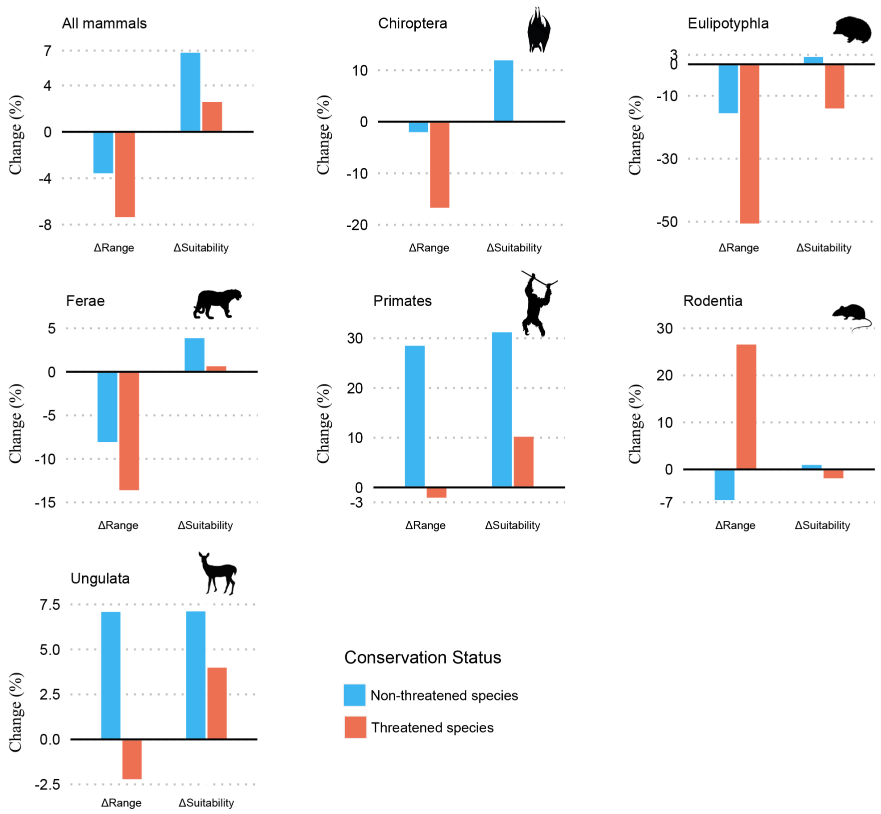

Fig D. Change (%) of range size (ΔRange) and suitability (ΔSuitability) between the last glacial maximum and the present-day, respectively, for all non-threatened and threatened species, for different taxa and minor clades occurring in the Indomalaya.

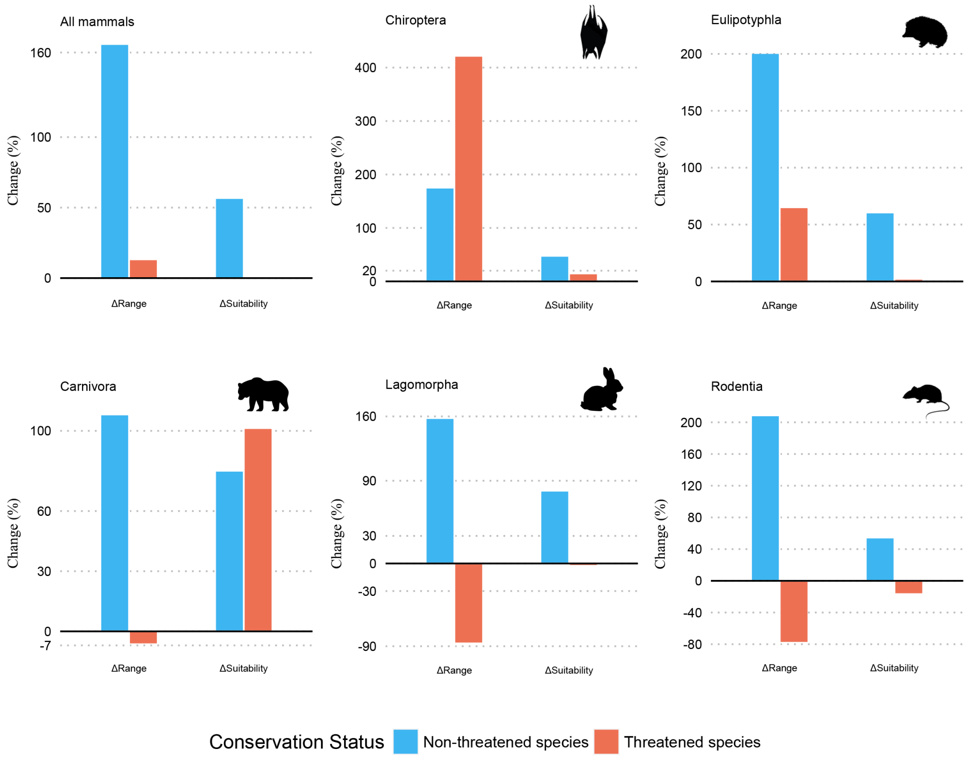

Fig E. Change (%) of range size (ΔRange) and suitability (ΔSuitability) between the last glacial maximum and the present-day, respectively, for all non-threatened and threatened species, for different taxa and minor clades occurring in the Nearctic.

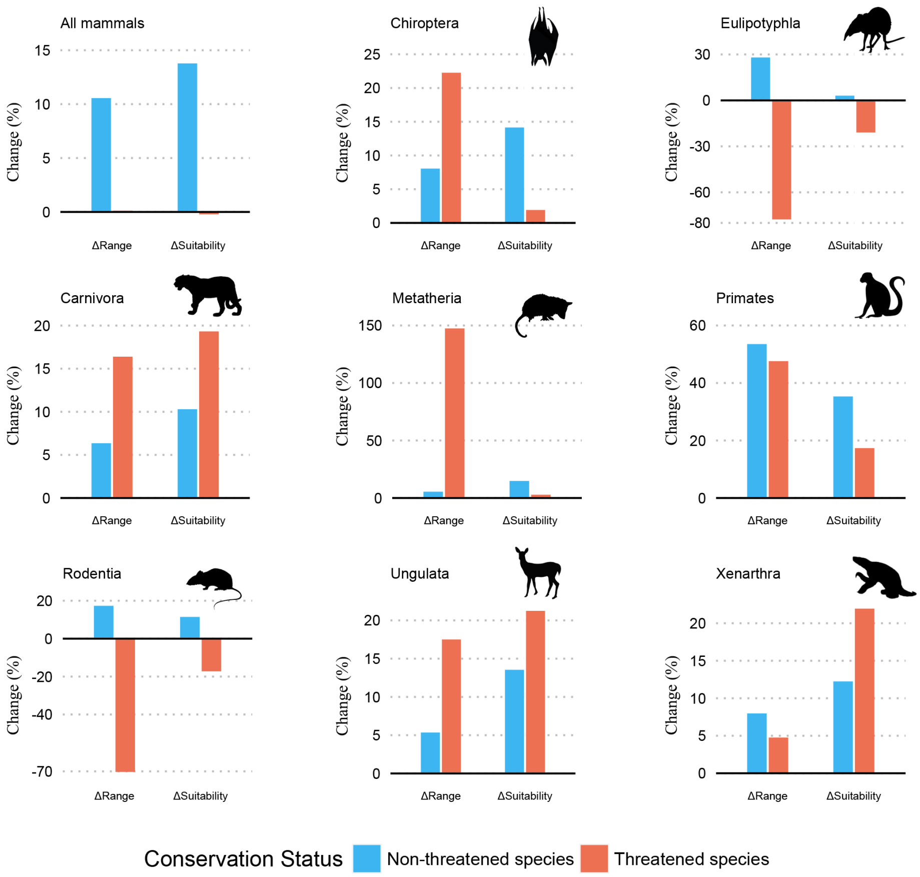

Fig F. Change (%) of range size (ΔRange) and suitability (ΔSuitability) between the last glacial maximum and the present-day, respectively, for all non-threatened and threatened species, for different taxa and minor clades occurring in the Neotropic.

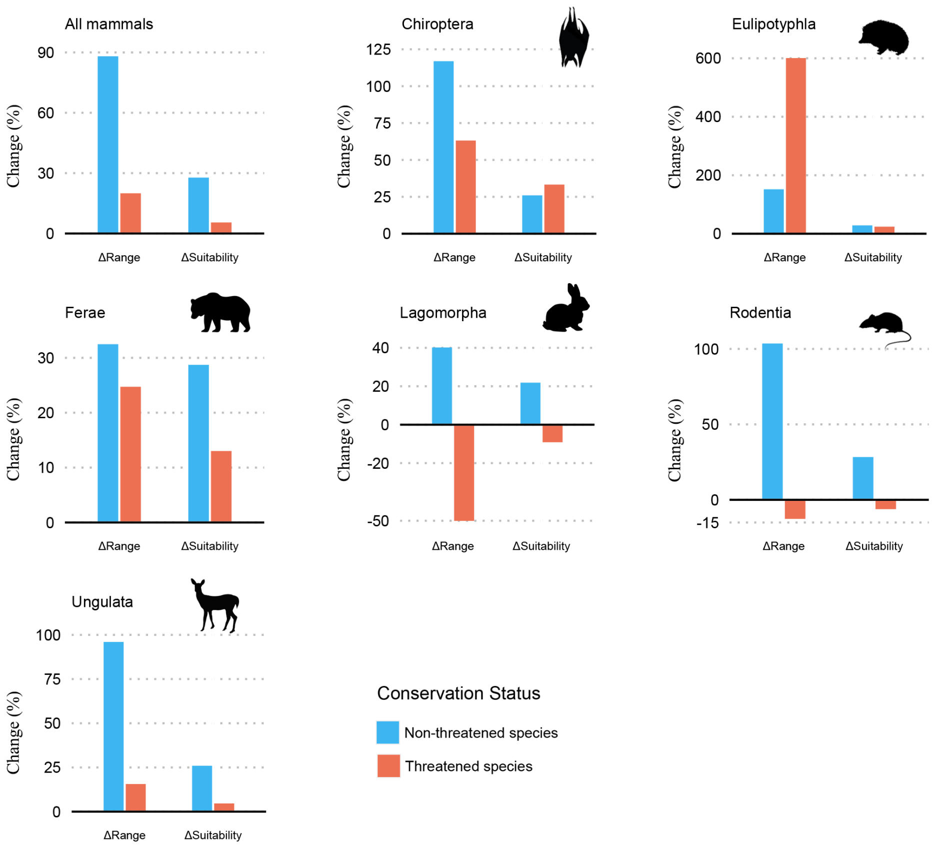

Fig G. Change (%) of range size (ΔRange) and suitability (ΔSuitability) between the last glacial maximum and the present-day, respectively, for all non-threatened and threatened species, for different taxa and minor clades occurring in the Palearctic.
